# Short-term effects of ambient air pollution and childhood lower respiratory diseases

**DOI:** 10.1038/s41598-017-04310-7

**Published:** 2017-06-30

**Authors:** Liyang Zhu, Xuhua Ge, Yaoyao Chen, Xinying Zeng, Wang Pan, Xu Zhang, Shuai Ben, Qi Yuan, Junyi Xin, Wei Shao, Yuqiu Ge, Dongmei Wu, Zhong Han, Zhengdong Zhang, Haiyan Chu, Meilin Wang

**Affiliations:** 1 Department of Environmental Genomics, Jiangsu Key Laboratory of Cancer Biomarkers, Prevention and Treatment, Collaborative Innovation Center for Cancer Personalized Medicine, Nanjing Medical University, Nanjing, China; 2 Department of Statistics, School of Economics, Nanjing University Of Finance & Economics, Nanjing, China; 3 Department of Emergency, Children’s Hospital of Nanjing Medical University, Nanjing, China; 4 Department of Genetic Toxicology, The Key Laboratory of Modern Toxicology of Ministry of Education, School of Public Health, Nanjing Medical University, Nanjing, China; 5 School of Public Health, Nantong University, Nantong, Jiangsu China

## Abstract

The association between air pollution and childhood respiratory disease is inconsistent. In the present study, we investigated a short-term effect of ambient air pollutants and daily childhood lower respiratory diseases (CLRD). Daily air pollutants, weather data, and CLRD data were collected from January 2014 to April 2015 (452 days) in Nanjing, China. Time-series regression and generalized additive models were used to assess the effects of air pollutants (PM_10_, PM_2.5_, NO_2_, SO_2_, O_3_, and CO) on CLRD. We observed that an interquartile range (IQR) increase in concentrations of PM_10_, NO_2_, and SO_2_ significantly increased the daily CLRD with 6 days cumulative effects (difference of estimates: 2.8%, 95% CI: 0.6–5.0%; 4.1%, 1.2–7.0%; 5.6%, 2.6–8.6%, respectively). However, no significant association was found in IQR concentrations of PM_2.5_, O_3_, and CO. Specifically, elevated PM_10_, PM_2.5_, NO_2_, and SO_2_ significantly increased the numbers of CLRD in cool season (3.6%, 1.5–5.7%; 2.4%, 0.3–4.5%; 4.9%, 2.9–7.0%; 6.3%, 3.7–9.0%, respectively). Additionally, the effect estimates of PM_10_, NO_2_, and SO_2_ in female and age >27 months were more pronounced than in male and age ≤27 months. This study suggested that short-term exposure to ambient PM_10_, NO_2_, and SO_2_ were associated with the increased CLRD numbers.

## Introduction

Air pollution is the important environment risk factor. Extensive data have indicated that air pollution is associated with a negative effect on human health^1^. WHO had proposed outdoor air pollution as the 14^th^ mortality risk factor in 2004. The ischemic heart disease and stroke were the main cause of deaths associated with air pollution. Additionally, pulmonary disorder also contributed to the disease mortality, among which acute lower respiratory diseases (LRD) was the common cause of children deaths under five years old^2^.

It is well known that the response of individual air pollutant exposure may be determined by age, sex, genetics, lifestyle and disease. The children are vulnerable to the detrimental effects of outdoor air pollutants on health. Several previous studies demonstrated significantly harmful effects of outdoor air pollutants on childhood lung function, e.g. exposure to particulate matter less than 2.5 µm in aerodynamic diameter (PM_2.5_), particulate matter less than 10 µm in aerodynamic diameter (PM_10_) and nitrogen dioxide (NO_2_) associated with reduced forced expiratory flows^3^. Recently, a few studies have found that acute and short-term exposure to outdoor air pollutants may increase the number of childhood allergic rhinitis, pneumonia, asthma symptoms^4, 5^. A recent meta-analysis including several cohort and cross-sectional studies suggested that exposure to traffic related PM_10_, PM_2.5_, NO_x_, sulfur dioxide (SO_2_), carbon monoxide (CO), ozone (O_3_) had increased frequency of childhood asthma and wheeze^6^. Potential mechanisms may be air pollution inducing allergic sensitization and oxidative stress, increasing the individual susceptibility to respiratory infection^7^.

At present, few association studies between air pollutants and Childhood LRD (CLRD) have been conducted in China^8, 9^. A recent study in Changsha (China) reported that early-life exposure to air pollutants can induce the higher childhood asthma^10^. Liu *et al*. collected the daily concentrations of PM_10_, NO_2_, and SO_2_ during the children life time (2006–2012) where they lived, and found that exposure to NO_2_ was associated with respiratory diseases in children^11^. Although the association studies of air pollutants and **C**LRD have been widely studied, the results remain inconsistent. Molter *et al*. found that outdoor air pollutants were not related with the multi-center childhood asthma in Europe^12^. Studies demonstrated that different NO_2_ exposure years of life were not associated with childhood asthma and asthma-related symptoms in the United States^13^.

It was worth to note that these studies had provided the effects of air pollutants on **C**LRD in location for public health assessments, however, the results might be affected by the local air pollutants levels, components and population susceptibility. It is important to study the effects of city air pollutants on **C**LRD numbers for the local city policy development^14^. Nanjing is a city located in the east of China, which is the provincial capital of Jiangsu province. With the development of economics, the concentrations of Nanjing’s air pollutants in central monitoring sites are above the nation’s levels. In the present study, we aim to conduct a short-term effect of ambient air pollutants, including PM_10_, PM_2.5_, NO_2_, CO, and O_3_, and daily CLRD in Nanjing, China.

## Material and Methods

### Data collection

Nanjing is a large city of Jiangsu province with a moderate subtropical climate (6597 km^2^). It had a population of 8.22 million in 2015. Daily CLRD data from 28 January 2014 to 24 April 2015 were obtained from Children’s Hospital of Nanjing Medical University, The Second Affiliated Hospital of Nanjing Medical University, Jiangsu Women and Clinical Health Hospital, Nanjing Jiangning Hospital, Nanjing First Hospital, Nanjing Gaochun People’s Hospital, Nanjing Maternity and Child Health Care Hospital. In this study, CLRD were comprised of doctor-diagnosed pneumonia (J18), bronchitis (J20), capillary bronchitis (J21), and acute asthma (J45), which were diagnosed based on the International Classification of Diseases 10th revision^15^.

Air pollution data including PM_10_, PM_2.5_, NO_2_, SO_2_, O_3_, and CO were obtained from China Environmental Monitoring Centre. These monitoring stations are not in direct vicinity to traffic or industrial sources, housing emissions such as oil, coal, or waste burning. Thus, the daily air pollution data of monitoring stations can represent the environmental air pollutants’ levels. To control the confounding effects of meteorological conditions on CLRD, we also obtained the data of daily weather data (relative humidity and mean temperature) from the Meteorological Bureau.

### Statistical analysis

Spearman’s correlation coefficient was applied to investigate the association between air pollutants and weather variables. We used the time-series regression model to explore the effects of each air pollutant (PM_10_, PM_2.5_, NO_2_, SO_2_, O_3_, and CO) on CLRD. We also used generalized additive model (GAM) to calculate the data^16^. Herein, the data of the respiratory symptoms followed the over-dispersed Poisson distribution, thus, we applied the quasi-Poisson regression in the GAM^17^. In order to control the potential confounding effect, firstly, we included a natural cubic smooth function of calendar time with 7 degrees of freedom (*df*) per year to remove unmeasured long-term and seasonal trends^18^; secondly, we included the natural smooth functions of the current-day relative humidity (3 *df*) and mean temperature (3 *df*) to exclude the weather confounding effects^19^; thirdly, we also included the indicator variable for “day of the week (DOW)” in the model. In this study, we also calculated the data in the cool period (November through April, 4 *df*) and warm period (May to October, 4 *df*) to separately analyze the effect of air pollutants on CLRD.

Further, we used single-pollutant model to explore the air pollution’s effect on CLRD with single lag days (lag0, 1, 2, 3, 4, 5, 6). To remove the potential misalignment of single day lag exposure, we considered the moving average exposure of multiple days (lag 0–1, 0–2, 0–3, 0–4, 0–5, 0–6) in additional analyses. We conducted the concentration-response relationship curves for the air pollutants concentrations with daily CLRD using a 3 *df* for the smoothing function in single-pollutant model. Herein, we performed the two-pollutants model to exam the stability of results.

The effect estimates were shown as the percentage change and 95% confidence intervals (CIs) in daily CLRD per interquartile range (IQR) increased of air pollutants’ levels. In this study, all models were conducted in R software (version 3.2.1) with mgcv package. All tests were two-sided, and *P* < 0.05 were considered as statistically significant.

## Results

### Data description

Table 1 presents the data of descriptive variables. There were 26,423 CLRD cases (male: 16,078, female: 10,344, sex unknown: 1; mean age: 26.6 months, Supplementary Table 1) during 28 January 2014 to 24 April 2015 (452 days), among which there were 5,551 bronchitis, 19,098 pneumonia, 1,560 capillary bronchitis, and 214 asthma cases. The daily average CLRD numbers were 58.5, and the maximum was 105.0. The daily average concentrations were 117.4 µg/m^3^ for PM_10_, 69.9 µg/m^3^ for PM_2.5_, 50.1 µg/m^3^ for NO_2_, 21.5 µg/m^3^ for SO_2_, 0.94 mg/m^3^ for CO, and 95.8 µg/m^3^ for O_3_. The daily average relative humidity and temperature were 72.5% and 15.1 °C, respectively.

**Table 1:** The summary of descriptive statistics in this study.

Variables	Mean ± SD	Minimum	25% quartile	Median	75% quartile	Maximum	Inter-quartile range
Daily CLRD numbers	58.5 ± 13.9	23.0	48.0	58.5	68.3	105.0	20.3
Air pollution (µg/m^3^)							
PM_10_	117.4 ± 56.5	17.8	76.0	108.9	147.8	389.2	71.8
PM_2.5_	69.9 ± 36.7	13.7	45.4	63.0	87.6	247.3	42.2
NO_2_	50.1 ± 18.8	14.2	36.2	47.5	62.3	118.1	26.1
SO_2_	21.5 ± 11.5	3.21	12.4	19.8	28.8	71.7	16.4
O_3_	95.8 ± 52.7	7.00	53.0	84.5	129.0	273.0	76.0
CO (mg/m^3^)	0.94 ± 0.89	0.35	0.70	0.89	1.11	2.45	0.41
Weather conditions							
Mean temperature (°C)	15.1 ± 8.2	−2.23	7.93	15.3	22.4	31.8	
Relative humidity (%)	72.5 ± 15.5	28.0	62.2	72.9	85.4	97.2	

CLRD, Childhood lower respiratory diseases.

As shown in Table 2, there were positively correlated among 6 air pollutants, except CO and O_3_ (r = −0.17, *P* = 0.001). In addition, air pollutants were also correlated with temperature and humidity.

**Table 2:** Spearman correlation between air pollutants and weather variables in Nanjing.

Variables	PM_10_	PM_2.5_	NO_2_	SO_2_	CO	O_3_	Mean temperature (°C)	Relative humidity (%)
PM_10_	—	0.87**	0.67**	0.75**	0.61**	0.24**	−0.07	−0.49**
PM_2.5_	0.87**	—	0.53**	0.56**	0.68**	0.22**	0.03	−0.21**
NO_2_	0.67**	0.53**	—	0.75**	0.53**	0.05	−0.22**	−0.46**
SO_2_	0.75**	0.56**	0.75**	—	0.47**	0.14**	0.28**	0.71**
CO	0.61**	0.68**	0.53**	0.47**	—	−0.17**	−0.22**	−0.13**
O_3_	0.24**	0.22**	0.05	0.14**	−0.17**	—	0.58**	−0.26**
Mean temperature (°C)	−0.07	0.03	−0.22**	−0.28**	−0.22**	0.58**	—	0.24**
Relative humidity (%)	−0.50**	−0.21**	−0.46**	−0.71**	−0.13**	−0.26**	0.24**	—

**P < 0.001.

### Main results

**Figure 1:**
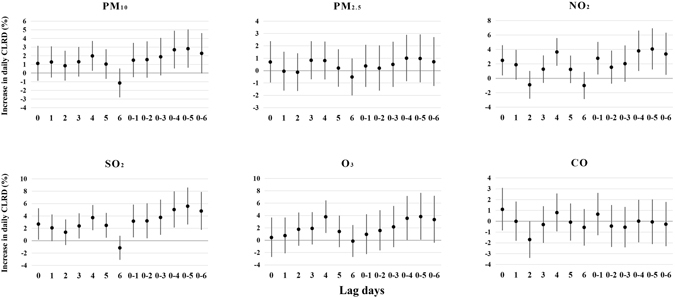
Estimated changes with 95% confidence intervals in daily CLRD (Childhood lower respiratory diseases) percentage deviations (%) associated with an interquartile range increase in PM_10_, PM_2.5_, NO_2_, SO_2_, O_3_ or CO concentrations with different lag days in single-pollu﻿tant model.

Single-pollutant models were applied to obtain the percentage change of CLRD associated with an IQR increase in air pollutants concentrations for single day lag (lag0, lag1, lag2, lag3, lag4, lag5, lag6) and multi-day lag (lag0–1, lag0–2, lag0–3, lag0–4, lag0–5, lag0–6) (Figure 1). An IQR increase in concentrations of PM_10_, NO_2_, SO_2_ at multi-day exposure were more significant associated with estimated increase in the numbers of CLRD than single day exposure. For lag0–5, we observed the highest significant association between PM_10_, NO_2_, SO_2_ and CLRD numbers (difference of estimates: 2.8%, 95% CI: 0.6–5.0%; 4.1%, 1.2–7.0%; 5.6%, 2.6–8.6%, respectively). However, no significant association was found in IQR concentrations of PM_2.5_, O_3_, and CO.

**Figure 2:**
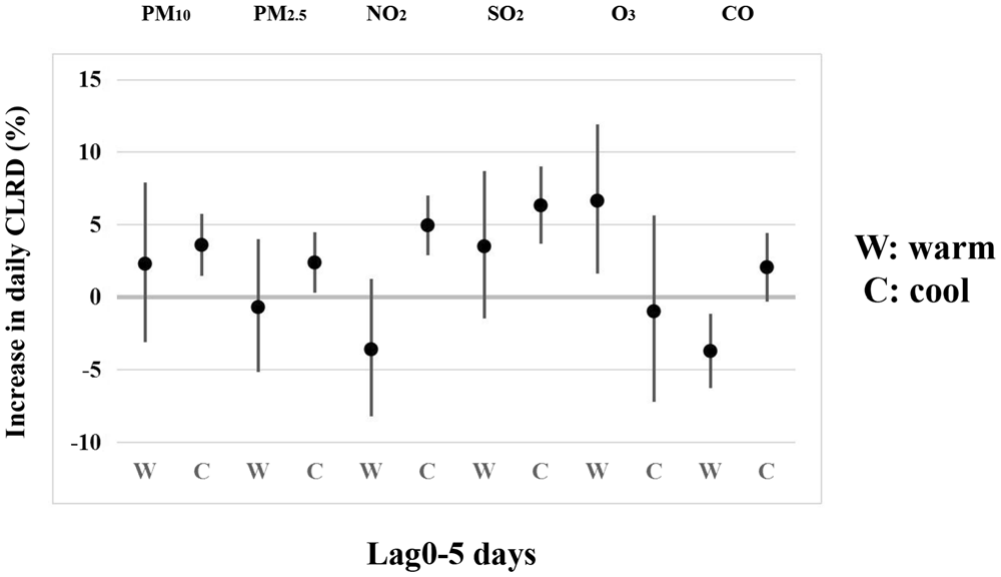
Estimated changes with 95% confidence intervals in daily CLRD percentage deviations (%) associated with an interquartile range increase in PM_10_, PM_2.5_, NO_2_, SO_2_, O_3_ or CO concentrations (lag0–5 days) by season using the single-pollutant model.

Further, we evaluated the effect estimates stratified by the season (warm and cool period). Figure 2 showed that the associations of PM_10_, PM_2.5_, NO_2_, and SO_2_ and CLRD numbers in cool period (3.6%, 1.5–5.7%; 2.4%, 0.3–4.5%; 4.9%, 2.9–7.0%; 6.3%, 3.7–9.0%, respectively) were more pronounced than in warm period. Additionally, we also explored the effect estimates of air pollutants on CLRD in the subgroup of sex, age and diseases (bronchitis, pneumonia, capillary bronchitis, and asthma cases) (Supplementary Figure 1–4). For PM_10_, we observed that the effect estimates were pronounced in female (4.0%, 0.6–7.6%), age >27 months (13.3%, 9.0–17.6%), and pneumonia (2.7%, 0.1–5.3%). For NO_2_ and SO_2_, results suggested that the significant effect was found both in male (3.9%, 0.4–7.5%; 4.4%, 0.7%, 8.2%) and female (5.2%, 0.8–9.8%; 7.8%, 3.1–12.7%). When stratified by age, besides PM_10_, an IQR increase in concentrations of PM_2.5_, NO_2_, SO_2_ and O_3_ exposure were significant associated with estimated increase in the numbers of CLRD (age >27 months) (12.2%, 8.5–16.0%; 13.2%, 7.8–19.0%; 9.9%, 4.5–15.6%; 12.9%, 6.2–19.9%, respectively). In the stratification of diseases, we found that PM_2.5_ was associated with positively increased risk of childhood asthma (28.2%, 3.2–59.3%), and NO_2_ was associated with increased risk of pneumonia (4.0%, 0.7–7.4%) and capillary bronchitis (19.7%, 8.1–32.6%), and SO_2_ was associated with increased risk of bronchitis (8.0%, 1.5–14.9%), pneumonia (4.3%, 0.9–7.8%) and capillary bronchitis (14.1%, 2.4–27.1%), and O_3_ was associated with increased risk of bronchitis (14.9%, 6.5–23.8%).

**Figure 3:**
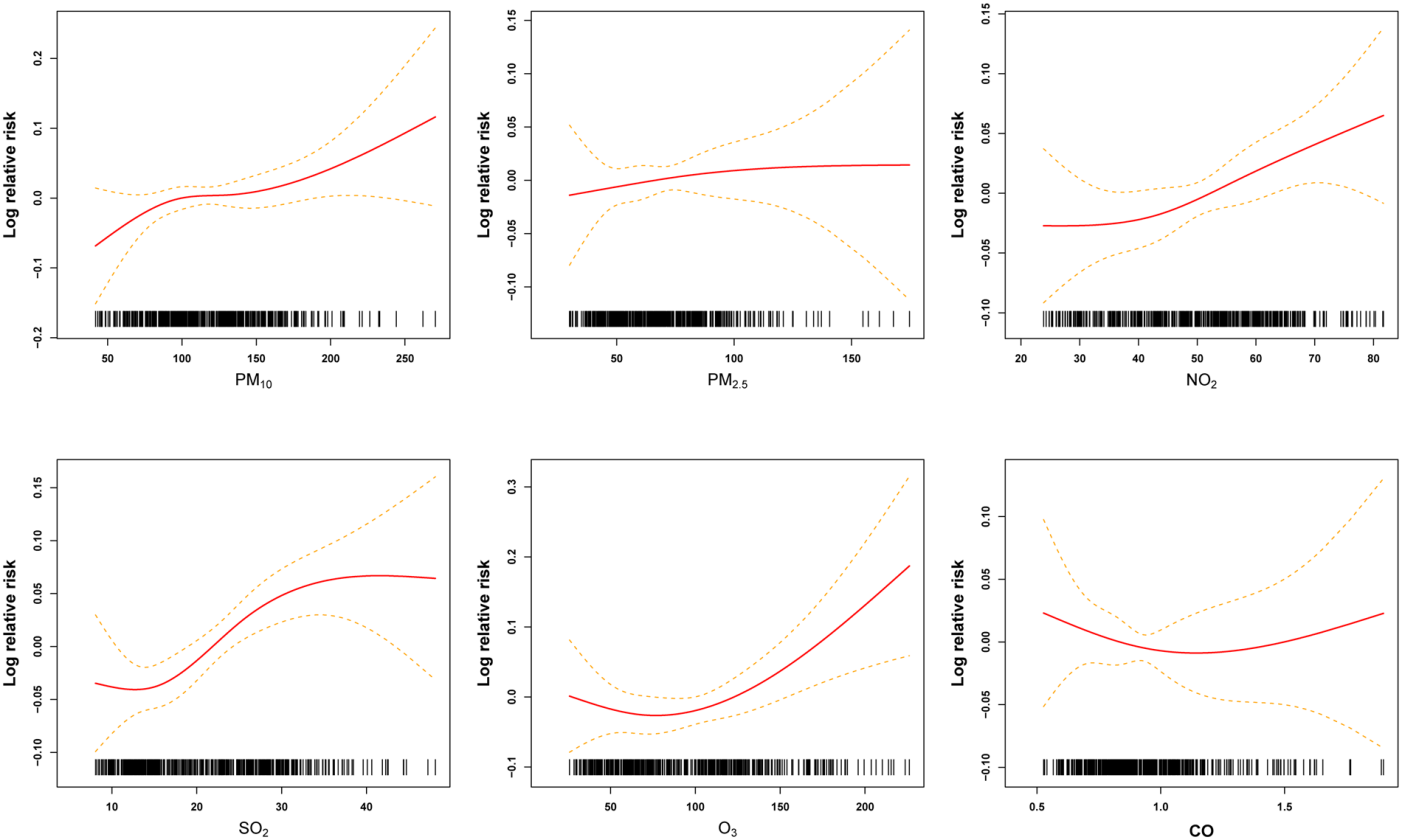
The concentration–response relationship curves for the air pollutants concentrations (lag0–5 day) with daily CLRD.

Concentration-response relationships of CLRD numbers with air pollutants also were performed (Figure 3). The curves for PM_10_, NO_2_ and O_3_ were similar and linearly positive and flat at higher concentrations. The effects of SO_2_ tend to be linearly positive with its concentrations, and appeared to be stable when concentration was beyond 40 µg/m^3^. However, the curves for PM_2.5_ and CO were linearly associated without any thresholds.

For lag 0–5, we also performed the two-pollutants model (Figure﻿ 4). Results suggested that the effect estimates of all six pollutants attenuated a little. However, when adjusted for PM_2.5_, two-pollutants model of PM_10_ produced the higher effect estimates for CLRD numbers than single-pollutant model (7.0%, 95% CI: 2.8–11.5%). Additionally, when adjusted for PM_10_, an IQR increase in PM_2.5_ pollutant concentration decreased CLRD numbers (−4.1%, 95% CI: −7.6–0.6%).

**Figure 4:**
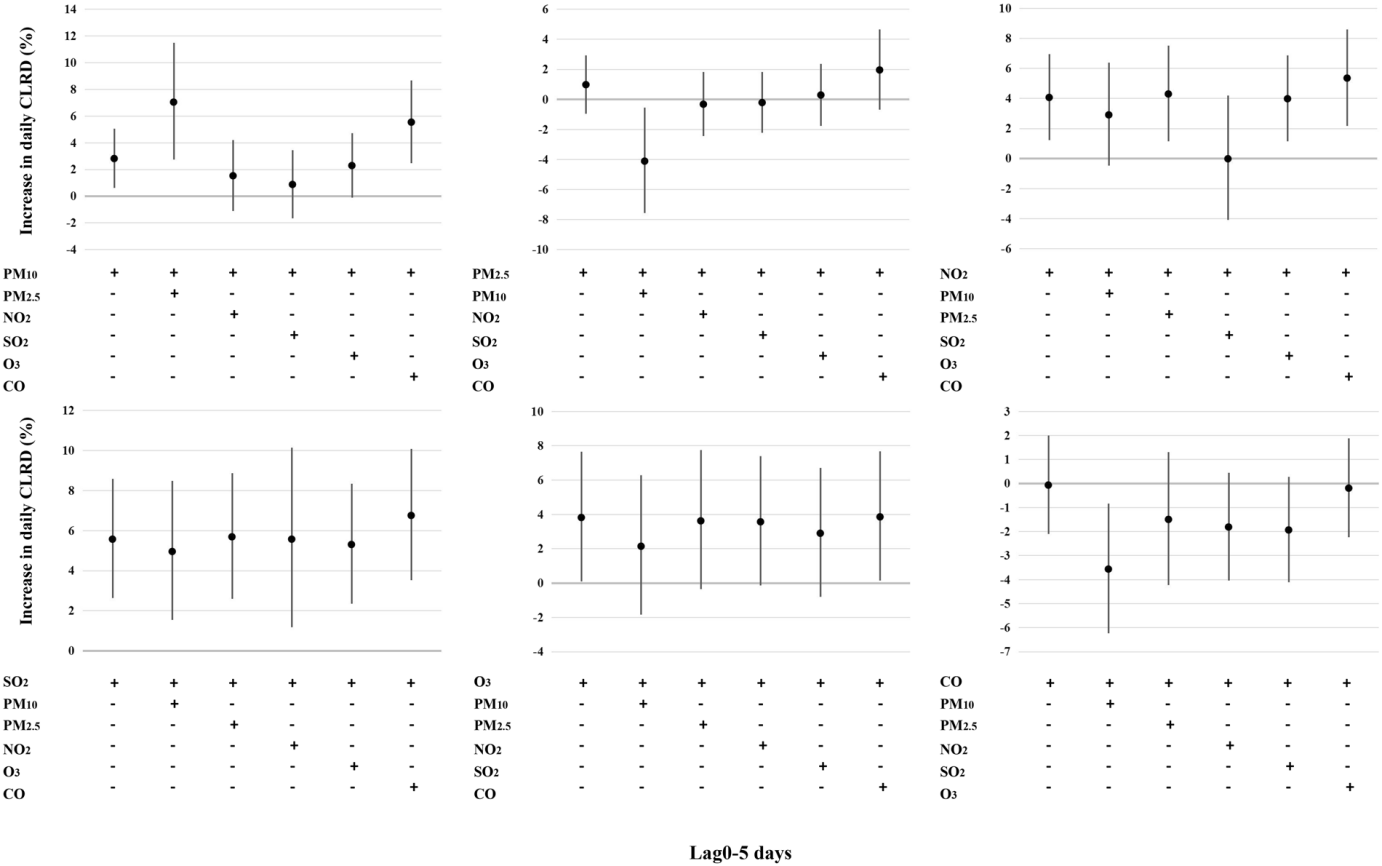
Estimated changes with 95% confidence intervals in daily CLRD percentage deviations (%) associated with an interquartile range increase in PM_10_, PM_2.5_, NO_2_, SO_2_, O_3_ or CO concentrations (lag0–5 days) in two-pollutants model.

## Discussion

The source of air pollution is mainly manufacturing industries, traffic, agriculture, forest fires, etc., which result in increasing air pollutants level. Exposure to outdoor air pollutants such as particulate matter, NO_2_, SO_2_ had the adverse effects on the airway responsiveness and affected the immune competent cells^20, 21^. Epidemiological association studies between the air pollutants and childhood respiratory health have been studied widely; however, results are conflicting. For example, in a prospective birth cohort, Molter *et al*. did not find any evidence of the effect on the association between PM_10_ and NO_2_ and childhood asthma or wheeze^22^. Similar to their study, a study in Norway suggested that exposure to NO_2_ was not associated with childhood asthma at age 9–11 years old^23^. European BAMSE study also observed no significant association between PM_10_ exposure and childhood asthma^24^. Nevertheless, Clark*et et al*. found the significant association between NO_2_, PM_10_, SO_2_ exposure and Canadian childhood asthma^25^.

Children exposure to air pollutants is vulnerable to suffering from pulmonary diseases, owing to a larger surface area of lung space than adult^26^. Exposure to higher air pollutants levels may induce the pathologic changes in childhood airway mucous and make children prone to the prevalence of chronic inflammation in respiratory system^27^. Many studies have investigated the association between air pollutants and CLRD, of which the majority suggested the effects of short-term exposure to air pollutants up to 4 or 5 days^28, 29^. Similarly, we also found a cumulative effect of PM_10_, NO_2_, and SO_2_ (lag0–5) on the short term effects with CLRD. Besides, we also investigated the effect estimates of PM_10_, NO_2_, and SO_2_ on CLRD by stratification of diseases (bronchitis, pneumonia, capillary bronchitis, and asthma cases). Some differences observed between PM_10_, NO_2_, and SO_2_ and diseases.

Exposure to PM_10_ can induce the lung function disorder of children. In this study, we observed that an IQR increase in PM_10_ daily concentrations increased the daily numbers of CLRD with 5, 6, or 7 days cumulative effects (lag0–4, lag0–5, lag0–6, respectively). Recently, a retrospective study also showed a positive association between the cumulative effects of 7 days (lag0–6) for PM_10_ and CLRD^15^. Further, we found the significant association between PM_10_ and pneumonia. A Jinan study revealed that PM_10_ were associated with increased number of Childhood pneumonia^30^. In 2010, Weinmayr and his colleagues conducted a systematic literature review to assess the effect of PM_10_ on respiratory health in children, which had provided the clear evidence of PM_10_ as the risk factor on childhood asthma symptom episodes^31^. However, we did not observe the similar association. It is possible that genetic background can contribute to the differences.

NO_2_ exposure can damage the lung function, and induce the change of allergic airway inflammation, and responses to allergens^32^. Similar to PM_10_, we found that exposure to NO_2_ was significantly associated with CLRD. The estimated effect for NO_2_ was consistent with many other studies^33, 34^. In Italy, Bono *et al*. found the adverse health effect of NO_2_ on the risk of emergency room admissions for childhood respiratory diseases^34^. A meta-analysis included seven countries also confirmed that exposure to NO_2_ was significantly associated with the asthma and wheezing of children aged 0–18^35^. In the present study, the significant effect estimate of NO_2_ on childhood pneumonia and capillary bronchitis was more pronounced than other diseases (bronchitis, and asthma).

SO_2_ is a well-known gas with strong irritant, and can induce the responses of respiratory system. A study showed that children living near a petrochemical complex with high SO_2_ exposure had significantly increased incidence of bronchitis, asthma and allergic rhinitis than low exposure group^36^. In addition, Samoli *et al*. also observed that exposure to SO_2_ (10 µg/m^3^) significantly increased the number of childhood asthma hospital admissions^37^. In this study, we also found that exposure to SO_2_ had a positive association with the numbers of CLRD, especially with childhood bronchitis, pneumonia, and capillary bronchitis.

Season was an important factor in modifying the association between air pollutants and population health effects^38^. In this study, we found the effects of air pollutants on CLRD numbers varied by season. Specifically, the effect estimates of PM_10_, NO_2_, and SO_2_ were positive associated with CLRD numbers in cool season. Chen’s findings also suggested that exposure to PM_10_ and NO_2_ had the largest effects in childhood asthma in cool season^39^. A system review and meta-analysis also proposed that children exposure to PM_10_, NO_2_, and SO_2_ have the stronger association of asthma-related hospital admission in warm season^40^. The exact seasonal reasons were still not clear, and might be related with the difference in air pollution levels, surrounding environment, or races.

Several studies have found that age and sex can influence the effect estimates of air pollutants on population health. In the present study, we explored the effect estimates of air pollutants on CLRD in the subgroup of sex and age. Results showed that the effect estimates of PM_10_, NO_2_, and SO_2_ in female were more pronounced than in male, which were consistent with previous study^41^. Similar results were observed in children with age >27 months. It was possible that children with age >27 months spent more time outdoors than age ≤27 months.

Herein, we should mention several limitations. Firstly, we obtained the data only from one city. Exposure to air pollutants effects may be influenced by person’s living and other factors. Results were difficult to spread to other cities. Secondly, we did not obtain the exactly individual exposure levels of air pollutants, thus, we merely calculated the average air pollution levels of local region to evaluate the estimate effects of air pollutants. Thirdly, we collected the useful data based on one and a half years data from the seven hospitals, which may not represent the entire populations. Besides, our study is the retrospective study, we cannot obtain the biochemical indexes of CLRD cases. Further larger studies should be conducted to comprehensively clarify the effects of air pollutants on CLRD. This study also had several strengths. Firstly, it was the first time to investigate the effect estimate of air pollutants on CLRD in Nanjing, China. Secondly, we used the time-series regression and GAM analysis to evaluate the effects of air pollution. Thirdly, although we obtained the data in a city, the results were consisted with many other studies^39, 40, 42^.

In summary, we found that the short-term exposure to ambient PM_10_, NO_2_, and SO_2_ were associated with increased CLRD numbers. It was worth to note that our data provided the limited information; therefore, further studies should be conducted to validate our findings.

## Electronic supplementary material

Supp data
