# Supplementary material for: Short-term effects of ambient air pollution and childhood lower respiratory diseases
Source: Sci Rep. 2017 Jun 30;7:4414. doi: 10.1038/s41598-017-04310-7 (PMC5493680; doi:10.1038/s41598-017-04310-7)

**Short-term effects of ambient air pollution and childhood lower respiratory diseases**

Liyang Zhu^a,b,十^, Xuhua Ge^c,十^, Yaoyao Chen^a,十^, Xinying Zeng^d^, Wang Pan^d^, Xu Zhang^d^, Shuai Ben^e^, Qi Yuan^a^, Junyi Xin^a^, Wei Shao^a^, Yuqiu Ge^a^, Dongmei Wu^a^, Zhong Han^b^, Zhengdong Zhang^a^,*, Haiyan Chu ^a^,*, Meilin Wang ^a^,*

^a^Department of Environmental Genomics, Jiangsu Key Laboratory of Cancer Biomarkers, Prevention and Treatment, Collaborative Innovation Center for Cancer Personalized Medicine, Nanjing Medical University, Nanjing, China; ^b^Department of Statistics, School of Economics, Nanjing University Of Finance & Economics; ^c^Department of Emergency, Children’s Hospital of Nanjing Medical University; ^d^Department of Genetic Toxicology, The Key Laboratory of Modern Toxicology of Ministry of Education, School of Public Health, Nanjing Medical University, Nanjing, China; ^e^School of Public Health, Nantong University, Nantong, Jiangsu, China

^十^Liyang Zhu, Xuhua Ge, Yaoyao Chen contributed equally to this work.

***Correspondence to:** Department of Environmental Genomics, School of Public Health, Nanjing Medical University, 101 Longmian Avenue, Nanjing 211166, China. Tel: +86 25 86868423; Fax: +86 25 86868499; Zhengdong Zhang, Email: [drzdzhang@gmail.com](mailto:drzdzhang@gmail.com); Haiyan Chu, Email: [chy_grape@njmu.edu.cn](mailto:chy_grape@njmu.edu.cn); Meilin Wang, Email: [mwang@njmu.edu.cn](mailto:mwang@njmu.edu.cn)

**Supplementary Table 1.** Basic demographic information of CLRD

| Variables | Numbers | Percent |
| --- | --- | --- |
| Total | 26,423 | |
| Sex |  | |
| Male | 16,078 | 60.8% |
| Female | 10,344 | 39.1% |
| Unknown | 1 |  |
| Age (mean±sd) (months) | 26.60±29.50 | |
| Disease |  | |
| bronchitis | 5,551 | 21.01% |
| pneumonia | 19,098 | 72.28% |
| capillary bronchitis | 1,560 | 5.90% |
| asthma | 214 | 0.81% |

**Supplementary Figure 1.** Estimated changes with 95% conﬁdence intervals in daily CLRD percentage deviations (%) associated with an interquartile range increase in PM_10_, PM_2.5_, NO_2_, SO_2_, O_3_ or CO concentrations (lag0-5 days) by age using the single-pollutant model.


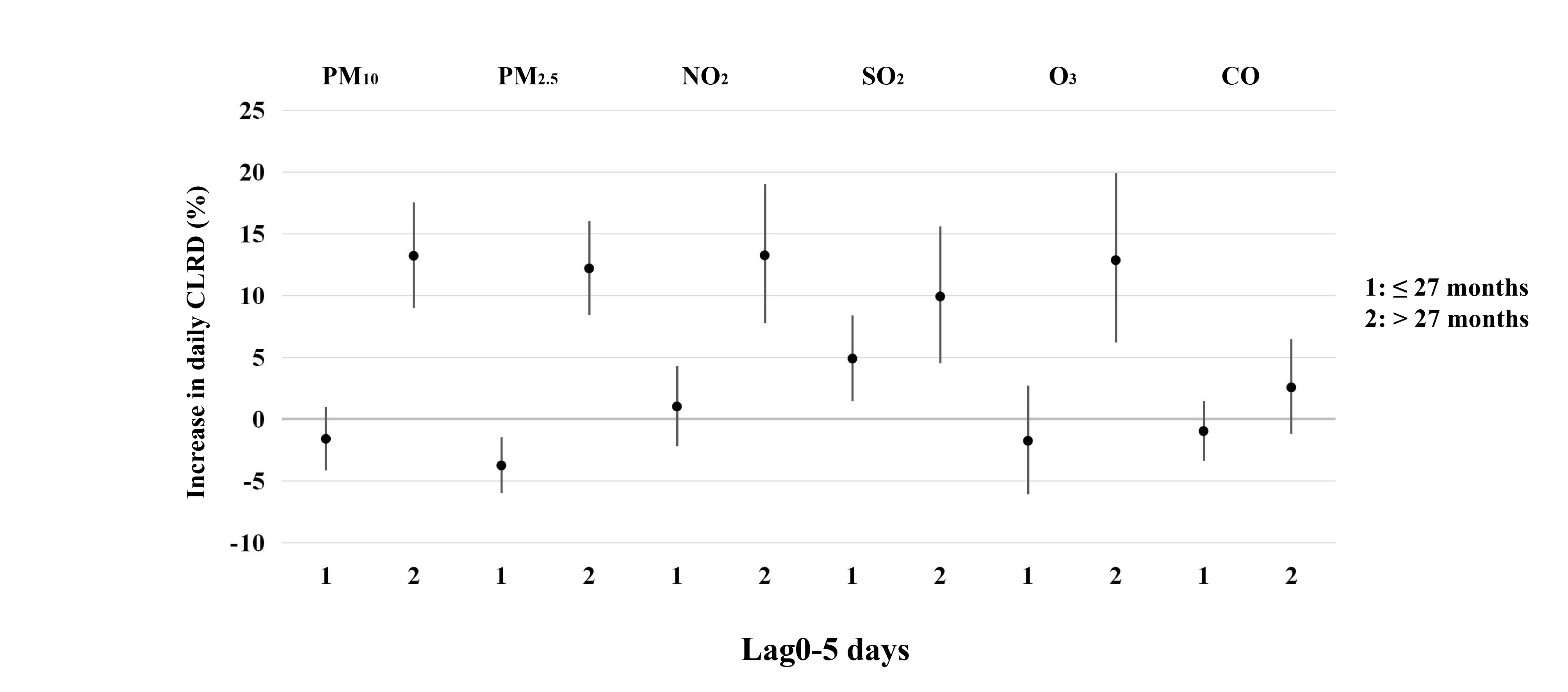


**Supplementary Figure 2.** Estimated changes with 95% conﬁdence intervals in daily CLRD percentage deviations (%) associated with an interquartile range increase in PM_10_, PM_2.5_, NO_2_, SO_2_, O_3_ or CO concentrations (lag0-5 days) by sex using the single-pollutant model.


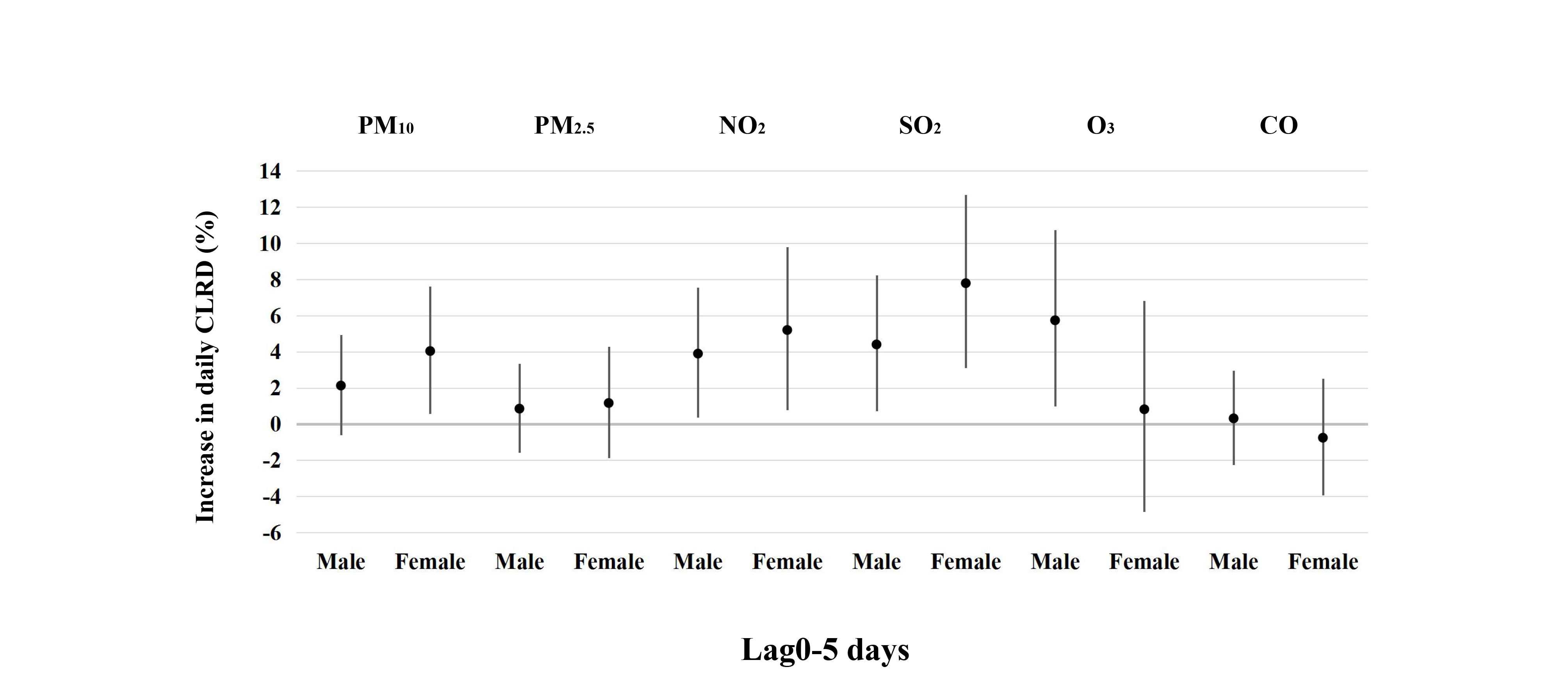


**Supplementary Figure 3.** Estimated changes with 95% conﬁdence intervals in daily CLRD percentage deviations (%) associated with an interquartile range increase in PM_10_, PM_2.5_, NO_2_, SO_2_, O_3_ or CO concentrations (lag0-5 days) by diseases using the single-pollutant model.


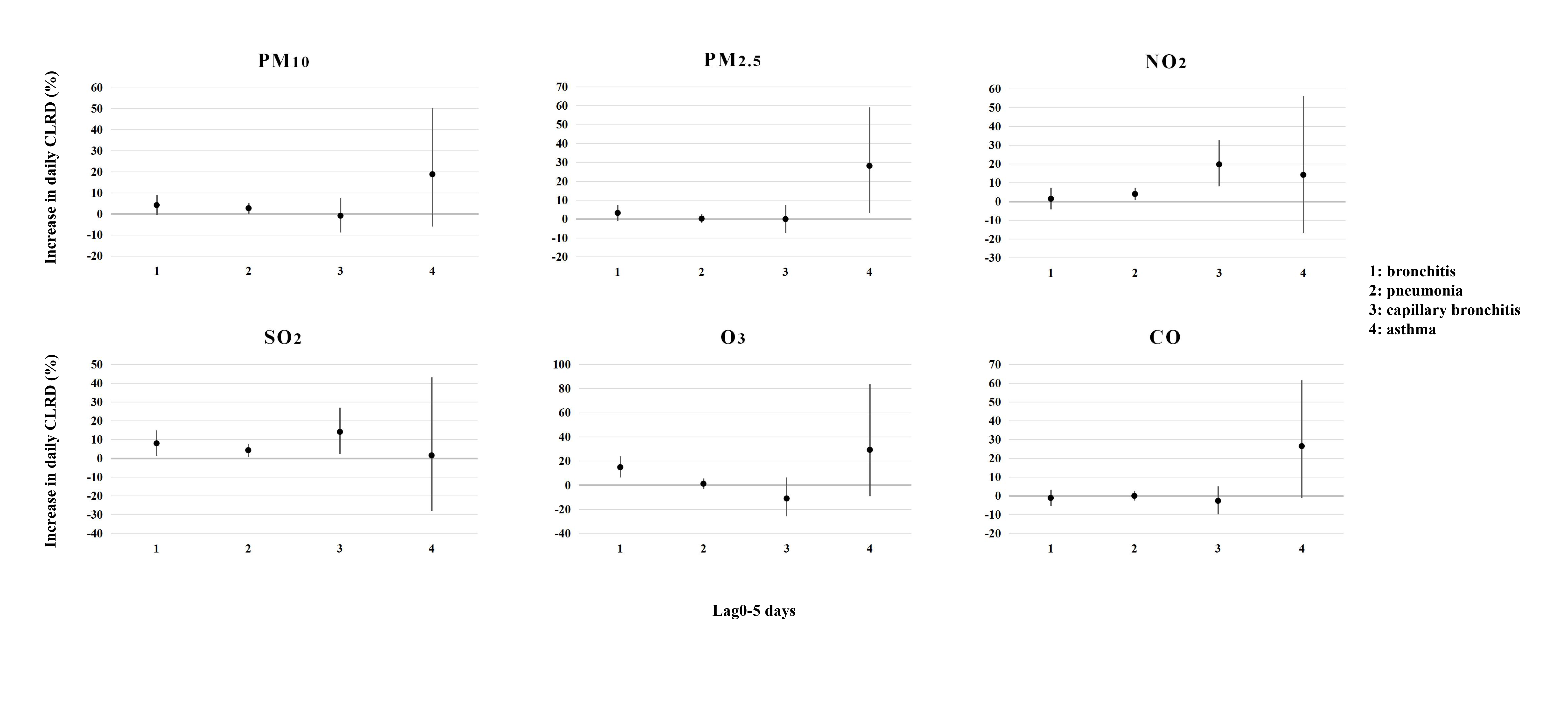

Supplement: Supplementary file 1 — Supp data [file 41598_2017_4310_MOESM1_ESM.doc]
